# Political determinants of Health: Lessons for Pakistan

**DOI:** 10.12669/pjms.303.5487

**Published:** 2014

**Authors:** Rashid Jooma, Guido Sabatinelli

**Affiliations:** 1 Rashid Jooma, FRCS (Eng), FRCSEd (SN), Department of Surgery, Aga Khan University, Karachi, Pakistan.; 2 Guido Sabatinelli, MD, WHO Representative in Tunisia, Tunis, Tunisia.

**Keywords:** Politics, Social Determinants of Health, Policy, Pakistan

## Abstract

There is much concern about the capacity of the health system of Pakistan to meet its goals and obligations. Historically, the political thrust has been absent from the health policy formulation and this is reflected in the low and stagnant public allocations to health. Successive political leaderships have averred from considering healthcare is a common good rather than a market commodity and health has not been recognized as a constitutional right. Over 120 of world’s nation states have accepted health as a constitutional right but the 1973 Constitution of Pakistan does not mandate health or education as a fundamental right and the recently adopted 18th constitutional amendment missed the opportunity to extend access to primary health care as an obligation of the State. It is argued in this communication that missing from the calculations of policy formulation and agenda setting is the political benefits of providing health and other social services to underserved populations. Across the developing world, many examples are presented of governments undertaking progressive health reforms that bring services where none existed and subsequently reaping electoral benefit. The political determinant of healthcare will be realized when the political leaders of poorly performing countries can be convinced that embracing distributive policies and successfully bringing healthcare to the poor can be major factors in their re-elections.

## INTRODUCTION

The lack of substantial progress towards the achievement of the Millennium Development Goals reflecting maternal and child health in Pakistan and the intractability of polio despite a vast global public health effort, has raised questions about the linkage of social service delivery and the political processes in the country.^1^^,^^2^ Analytical observation has shown that the strategic vision which underpins good governance is not effective in positively influencing the health system of Pakistan.^3^ It is now being increasingly appreciated that the ultimate determinant of health in communities and nations is political: the politics of the policy makers, the politics of the healthcare providers and the politics of the population. Dr. Halfdan Mahler, the Director General of WHO through the seventies, once said, “Health is politics and politics is health on a large scale. If you want to move health public policies in a big manner, then you have to have the political dynamite that is necessary”.^4^ This growing recognition in the public health community was reflected in the World Conference on Social Determinants of Health, held in December 2010 and culminating with the Rio Political Declaration^5^, which stressed the importance of policy and orientation of politics in health outcomes of populations.

***Politics of Health Policy: ***Politically progressive governments promote progressive policies, which aim towards reducing the adverse impact of social inequalities on health and recognize that social justice is the foundation of public health. Prof Navarro of Johns Hopkins conducted a survey of a set of OECD countries for the link between political ideologies and health policy, to see how politics determines public policy and thus affects health outcomes in populations. He classed the countries into 4 political traditions ranging from social democratic to authoritarian, based on their own identification and their implementation of redistributive policies. His analysis made an empirical link between politics and policy and health outcomes. An important finding was that the implementation of policies aimed at reducing social inequalities seems to have a salutary effect on a population’s health. Specifically, health indicators such as infant mortality were better in countries that had been governed by pro-redistributive political parties.^6^^,^^7^

In Pakistan, historically, the political thrust has been absent from the formulation of health policy and this is reflected in the low and stagnant public allocations to health over time. Conversely, the out-of-pocket expenditure for health in Pakistan is among the highest in the world and is considered a major contributor to poverty. This is irrespective of the stated political philosophy of the party in power or the commitments of their election manifestos, suggesting that pronouncements of adherence to principles of distributive justice remain unfulfilled. Successive political leaderships have averred from considering healthcare as a common good rather than a market commodity and health has not been recognized as a constitutional right.

***Health as a Fundamental Right: ***Article 25 of the Universal Declaration of Human Rights^8^ of 1948 states that "Everyone has the right to a standard of living adequate for the health and well being of himself and his family..." while the constitution of the World Health Organization also declares that “The enjoyment of the highest attainable standard of health is one of the fundamental rights of every human being”.^9^ Over 120 of world’s nation states have accepted health as a constitutional right, recognizing that an effective health system is a core institution of any society as much as a fair justice system or a democratic political dispensation. It is only through strong health systems that the right to the highest attainable standard of health can be achieved. The 1973 Constitution of Pakistan did not mandate health or education as a fundamental right. Thus, provision of public health or curative care to citizens in need is not legally enforceable and a major failure of the state is the lack of universal access to reasonable safety nets in health. Another downside to the absence of legal mandate for health is that many health programs are formulated and funded to launch but then suffer neglect and handicaps as they do not suit an incoming administration and there is no statuary protection or place for judicial intervention. As part of the recent 18th amendment, Primary Education was inserted as a fundamental right but the health system of Pakistan has been dealt a double blow: The unplanned devolution of health along with abolition of the Federal Health Ministry is having adverse effects on the health system which could be wide ranging and long lasting and secondly, an excellent opportunity to extend access to Primary Health Care as an obligation of the state and a right of citizenship has been lost. Many of our healthcare seeking public are deprived of services due to lack of access and the fact that the prosperous sections of the population enjoy a reasonably good health status implies that the technical means to achieve good health do broadly exist in our country today. In fact, for the vast majority, the key barriers to good health are not the lack of technology but poverty and health system inequity that fails those in greatest need.

***Spotlight on Rwanda: ***The recent gains of Rwanda in health and education have caught much attention as it continues to recover from the 1994 genocide and its infrastructural devastation. Even today, it has a GDP per capita of $1100 and over 60% of the population lives below the poverty line but it is one of the few low-income countries on track to meet its Millennium Development Goals.^10^ This has happened consequent to implementation of innovative health service initiatives such as payments to state health workers for performance related to maternal and child health indicators.^11^ President Paul Kagame is widely recognized as authoritarian and even ruthless but not corrupt and his electoral passage has been facile owing to him having made social development a priority. The corruption of “bottom billion” ruling elites that impede the transformation of public monies into public services is absent and he has backed reform with resources so that total health spending has risen from $9 per person in 2000 to $34 in 2006.^12^ A health insurance scheme that offers cover for basic health conditions extends to 92% of the population and this with an annual premium of $2. Rwanda’s health financing scheme has vastly increased utilization of health services and is improving the nation’s health indicators. A recent study found that the incidence of catastrophic health expenditures amongst the country’s insured was 4 times less than in households with no cover.^13^ Rwanda’s real success has been in its transparent and prudent use of aid so as to be a magnet for donors and global health agencies and up to 53% of the total health expenditure is from foreign donors.^14^

***Expenditures for Health in Pakistan: ***In the past decade, there has been an accelerating trend in aid for health. Development assistance to health rose from US$5.6 billion in 1990 to $10.7 billion in 2000.^15^ In that year the Millennium Development Goals were set by the UN and Official Development Assistance (ODA) for health has subsequently rapidly increased to US$26.8 billion in 2010.^16^ Pakistan has not been able to capitalize on this largesse and only attracts donor funding of around USD 60 million annually, which accounts for about just 2% of our national health expenditure.^17^ At this level, Pakistan lags behind other low-income countries where donor assistance averages 14% of health spending and is as much as 22% in the case of Bangladesh. The adverse impact of these low contributions on the health system is compounded by the miserly allocations to health by the federal and provincial governments: in the year 2009-10, government allocations to health were US$1 billion in a GDP of US$175 billion i.e. 0.57%.^18^ The yawning gap between what our state allows annually for the health of each citizen, US$6, and what the WHO suggests that governments in developing countries spend to ensure basic essential health services, US$35-50, can only be bridged in the near term by increasing health funding of the public sector by 2 or 3 times along with working with donors to enhance support. The donor nations and agencies for their part have increasingly focused on “aid effectiveness” and expect recipient nations to ensure transparency, financial probity and accountability in utilization of aid.^19^ Project aid is now accompanied by complex monitoring and evaluation regimes and policy benchmarks are attached to serve as incentives for policy improvements. However, where political will is weak, there are severe limitations to what aid can do to leverage improved governance and in the donor community the perception of misappropriation and misuse of aid for health by an unholy nexus of state functionaries and political bosses in Pakistan is widely held. The cause is not helped by the manifest lack of enthusiasm in the political classes for enhancing state revenues by taxing agricultural incomes and support for greater documentation of commercial activities. Donors like to see themselves as assisting those who are earnest in helping themselves but Pakistan’s tax to GDP ratio of 8.5 is one of the lowest in the world and is scarce comfort to the development partners.

***Health as a Social Good: ***Government spending on health from domestic sources is an important indicator of a government's commitment to the health of its people and reflects the priority that the policy makers attach to provision of social goods.^20^ Such prioritization must be borne out of a political commitment that is robust enough to see off competing and external demands. It is instructive to compare the commitment to health by successive governments of Cuba, Iran and Pakistan as revealed by financial outlays. When state health expenditure is prioritized, out-of-pocket expenditure for private services is reduced and health indicators are improved (Table-I). In the early 1990s, demands of the IMF’s Structural Action Program were not resisted by the Pakistan Government and led to cuts in health budgets, introduction of user charges along with availability of financing and incentives for private health establishments.^21^ This led to a significant reversal of policy and loss of gains in Primary Healthcare flowing from the 5^th^ and 6^th^ Five year plans when the goals of the 1978 Alma Ata Declaration were embraced and the network of Basic Health Units vastly expanded. The thrust of this policy shift from preventive to curative care, led to the exponential growth of an unregulated for-profit healthcare market with a concurrent “run down” of the public health system. It is scant comfort that the IMF and the bilateral and multilateral development agencies have in the past decade, changed their approach to centre-staging the social needs of the underserved and the vulnerable, encouraging governments to foster programs that are pro-poor and contributing to human development.^22^ Too much profit for health entrepreneurs (who are often state health employees) and others that benefit from the flourishing private services and the weaknesses of the state system has passed under the bridge and turning the tide will require major progressive political reform. 

***Electoral Returns***: Across the developing world, many examples are available of governments undertaking progressive health reforms that bring services where none existed and subsequently reaping electoral benefit. Mexico’s Revolutionary Party (PRI) was in power for over 70 years until 2000 when The National Action Party (PAN) was elected on a platform of change. By law, health was made a constitutional right and conditional cash transfers introduced for adherence to several education, health and nutrition interventions. The National Health Program was announced in 2001 and as envisaged in it, Popular Health Insurance ensured universal access in 2004.^23^ The *PAN* again won the election of 2006. In Thailand, Thaksin Shinawatra had been a very popular Prime Minister owing to the effectiveness of his policies in reducing rural poverty and the introduction of the country’s first universal healthcare program, well known as the 30-baht scheme.^24^ This was a revolution and increased access to healthcare from 60 to 96% of the population. Despite facing an adverse political circumstance that led to his exile, Takshin remains a hugely popular figure and the election of his sister was widely seen as a proxy election. Two of the most popular Latin American presidents in recent times have been Lula da Silva of Brazil and Hugo Chavez of Venezuela. Both have espoused distributive policies and successfully bringing healthcare to the poor had been major factors in their re-elections. (According to one academic study, the successes of the Barrio Adentro program in 2003 and 2004 may have "crucially inﬂuenced" Chavez’s 59% to 41% victory in the Venezuelan referendum).^25^ In each of these nations, a major public health initiative had been undertaken to improve access to preventive and promotive services with enough impact to capture the public imagination.^26^^,^^27^ A health financing scheme for those in the lower bands of poverty, subsidy for inpatient care and compensation for traumatic injury are some such initiatives, which have been possible within the constraints of resources in developing nations.^28^

**Table-I:** Comparison of health expenditure between Cuba, Iran and Pakistan

Countries	General Government Expenditure on Health as % of total Government Expenditure		Government Expenditure on Health per Capita in USD		Private Expenditure on Health as % of Total Expenditure		Life Expectancy at birth (Years)	IMR per 1000 live births
	2002 → 2012		2002 → 2012		2002 → 2012			
Cuba	11.2	14.0	171.7	573.8	11.9	5.3	78	4
Iran	9.4	10.1	43.9	137.6	59.4	60.3	73	15
Pakistan	3.0	2.5	4.3	8.0	71.2	73.0	67	69

Source: WHO Global Health Observatory Data Repository.

GDP= Gross Domestic Product, USD= US Dollar, IMR=Infant Mortality Rate.

## CONCLUSION

What are the lessons for us from these disparate and diverse political systems? How can the health needs of the people be transformed into political reform? The world examples of electoral gains from provision of health services to the poor must be underscored. The political classes need to be convinced that security of tenure in office and renewing mandates from the electorate is more reliably contingent on demonstrated performance in provision of social services to those in need rather than the vagaries of the traditional rough and tumble of politics in Pakistan. It is shortsighted of the politicians to degrade the state’s health institutions in the scramble for power and patronage to fuel their political machines with a narrow focus on re-election rather than public service. The evidence for the societal benefits of investing in public health are widely available and armed with this rich body of evidence, it is the duty of physicians to lead civil society in influencing policy formulation to conform to the principles of distributive justice.
